# The LBX2‐AS1–miR‐491‐5p–PKM2 Positive Feedback Loop Promotes Radiation Resistance of Esophageal Cancer by Enhancing Glycolysis

**DOI:** 10.1155/bmri/1876375

**Published:** 2025-11-13

**Authors:** Fuhui Zhang, Zhiwu Wang, Xuemin Yao, Qingyu Zhang

**Affiliations:** ^1^ Department of Gastroenterology, Tianjin Medical University General Hospital, Tianjin, China, tjmugh.com.cn; ^2^ Department of Radiation and Chemotherapy Division, Tangshan People′s Hospital, Tangshan, China, tsrmyy.cn; ^3^ Department of Radiation and Chemotherapy Division, North China University of Science and Technology Affiliated Hospital, Tangshan, China, ncst.edu.cn; ^4^ Department of Radiation Division, The Fourth Affiliated Hospital of Hebei Medical University, Shijiazhuang, Hebei Province, China

**Keywords:** esophageal cancer, glycolysis, LBX2-AS1, PKM2, radiotherapy

## Abstract

**Background and Objective:**

Radioresistance is a significant factor affecting the therapeutic efficacy of radiotherapy. This study is aimed at investigating the molecular mechanism by which LBX2‐AS1 regulates pyruvate kinase M2 (PKM2) to influence radioresistance and its potential as a biomarker for radioresistance in esophageal cancer.

**Methods and Key Findings:**

Radioresistant sub‐cell lines, KYSE150R, were established in KYSE150 cells, and PKM2, cyclin D1, HIF‐1*α*, and LBX2‐AS1 levels were elevated in KYSE150R. Upregulated and downregulated PKM2 sub‐cell lines were established. Upregulating PKM2 increased the PKM2 level in the nucleus and increased levels of HIF‐1*α*, cyclin D1, and LBX2‐AS1. The knockdown of PKM2 showed the opposite result. Downregulated LBX2‐AS1 sub‐cell lines were established. Downregulation of LBX2‐AS1 decreased cell proliferation, glycolysis, cell cycle progression, and radioresistance, along with a reduction in cyclin D1, HIF‐1*α*, and PKM2 levels. The dual‐luciferase reporter system was used to verify that LBX2‐AS1 directly binds to miR‐491‐5p, and miR‐491‐5p directly binds to the 3 ^′^UTR of PKM2 mRNA. Downregulation of miR‐491‐5p in sh‐LBX2‐AS1 cells could increase cell proliferation, cell cycle, glycolysis, and radiation resistance. The LBX2‐AS1 level in serum of patients with esophageal cancer was detected, and its clinical relevance was analyzed. Results showed that high LBX2‐AS1 levels correlated with worse disease control, increased lymphatic metastasis, and poorer overall and progression‐free survival.

**Conclusion and Clinical Implications:**

LBX2‐AS1‐miR‐491‐5p‐PKM2 positive feedback loop enhances the radioresistance of esophageal cancer cells by altering the cell cycle and enhancing glycolysis. High level of LBX2‐AS1 in serum was correlated with worse DCR, lymphatic metastasis, worse overall survival, and progression‐free survival.

## 1. Introduction

According to Global Cancer Statistics 2020, esophageal cancer (EC) is one of the most common cancers in the world, as it has the eighth highest incidence and is the sixth leading cause of cancer‐related mortality [1]. The annual mortality of EC is about 100 per 100,000 people, and China has the highest incidence and mortality rate of EC in the world; the 5‐year overall survival rate is < 30%. At present, the mainstream treatment of EC includes surgical resection, targeted therapy, immunotherapy, radiotherapy, and chemotherapy [2]. Due to the lack of specific early symptoms and not obvious signs, most patients with EC are already in the advanced stage of cancer at the time of treatment, and the therapeutic effect of surgical resection is poor [3]. Radiotherapy is essential for the treatment of patients without surgical conditions and locally advanced EC [4]. Although concurrent chemoradiotherapy has become the standard treatment for advanced localized EC, radiation resistance generated during radiotherapy has become one of the important factors for the poor prognosis of patients with EC [5]. Then, 40%–60% of patients with radiotherapy for EC may suffer from radiotherapy insensitivity or recurrence [6]. Therefore, it is necessary to search for markers that can help accurately predict radiotherapy sensitivity, so as to develop new detection targets and treatments for EC.

Cancer cells have a faster rate of growth and metabolism than normal cells. Even in the condition of sufficient oxygen, cancer cells still use glycolysis as the main way of energy supply, which is called the “Warburg” effect, that is, aerobic glycolysis [7]. Aerobic glycolysis is an important factor affecting radiotherapy sensitivity. Recent studies have found that the intermediate of the aerobic glycolysis pathway in cancer cells can remove free radicals and reactive oxygen species (ROS), inducing cancer cell resistance to radiation therapy [8, 9]. Therefore, rate‐limiting enzymes that regulate glycolysis are key targets for increasing radiosensitivity by inhibiting the glycolytic pathway.

Since EC tissue specimens cannot be obtained from radiotherapy patients, serum markers that can reflect the level of glycolysis have high clinical value. Long noncoding RNAs (lncRNAs) are a class of RNA that are longer than 200 bp in length, lack an open reading frame, do not code for proteins, are usually composed of multiple spliced exons, are transcribed by RNA polymerase II, and have histone modifiers similar to those that encode proteins [10]. LncRNAs play an important role in the occurrence and development of various cancers and could control the expression of target genes through transcriptional and posttranscriptional interactions with various RNA molecules or proteins [11, 12]. These abnormally expressed lncRNAs serve as biomarkers for cancer diagnosis and treatment. Studies have shown that lncRNA is also involved in the regulation of radiotherapy resistance [13]. LBX2 antisense RNA 1 (LBX2‐AS1) is a member of the lncRNA. It is located in the chr2p13.1 region and is 1786 bp in length. There is evidence that LBX2‐AS1 is associated with the progression of EC [14], but the relationship between LBX2‐AS1 and radiotherapy sensitivity has not been reported. Our study found that LBX2‐AS1 was detectable and stable in serum samples and is involved in the regulation of glycolytic pathways. This study explored the relationship between LBX2‐AS1 and radiotherapy sensitivity of EC and verified the mechanism.

## 2. Materials and Methods

This study was approved by the Tianjin Medical University Cancer Institute and Hospital, and prior informed consent was obtained from all patients.

### 2.1. Patients and Samples

Informed consent from patients with EC undergoing radiotherapy at the Fourth Hospital of Hebei Medical University between 2016 and 2019 was obtained. The pretreatment blood samples and clinical data of the patients were collected. The blood samples were centrifuged at low speed within half an hour to obtain the upper layer serum, which was then stored at −80°C in the refrigerator. The ethics of the study were reviewed by the Tianjin Cancer Hospital. The inclusion criteria for patients were those who had not undergone surgery or chemotherapy prior to radiotherapy. During the study period, a total of 78 patients met the inclusion criteria, and 72 of them completed follow‐up assessments, resulting in a follow‐up rate of 92.3%, with a median follow‐up of 27 months. The average age was 67.41 ± 2.96, with a male‐to‐female ratio of 48/24. Inclusion criteria are as follows: (1) patients who only received radiation therapy and did not undergo surgery or chemotherapy and (2) patients with available long‐term follow‐up data. Exclusion criteria include (1) radiotherapy patients who had undergone surgery or chemotherapy and (2) patients with a follow‐up time of less than 1 month.

### 2.2. Cell Culture

The human EC cell line KYSE150 was preserved by Tianjin Cancer Institute. The radioresistant EC cell line was derived through continuous irradiation and subsequent culturing and passaging using a medical linear accelerator. The cells were cultured in RPMI‐1640 medium (Gibco) supplemented with 10% fetal bovine serum (FBS, PAN, Adenbach, Germany) and maintained in a 37°C, 5% CO_2_‐humidified incubator (Thermo Fisher).

### 2.3. Colony Formation Assays

A colony formation assay was performed to assess the radioresistance ability of the cells. In the colony formation assay, the treated cells were seeded at a density of 1 × 10^3^ cells per well in a six‐well plate and cultured for 7–14 days. The cells were fixed using polyformaldehyde and stained with 0.5% crystal violet. A cluster consisting of 50 or more cells was considered a colony.

### 2.4. Metabonomic Analysis

After cell collection, liquid nitrogen extraction was performed followed by grinding. The cells were then subjected to methanol extraction (4°C) and sonication. The supernatant was collected after centrifugation and used for further analysis. For chromatographic separation, an Acquity HSS T3 column (1.8 *μ*m, 100 × 2.1 mm; Waters) was employed with linear gradient elution using Solvent A (0.1% formic acid in water) and Solvent B (0.1% acetonitrile). The raw data obtained were processed using Compound Discoverer 3.0 software (Thermo Fisher Scientific, United States). Further matching was performed against the METLIN (http://metlin.scripps.edu) and HMDB (http://www.hmdb) databases.

### 2.5. Cell Proliferation Assays

The MTS assay was performed to evaluate the cell proliferation capacity. In the MTS assay, the treated cells were seeded at a density of 1 × 10^3^ cells per well in a 96‐well plate. After the cells adhered to the plate, 20 *μ*L of MTS reagent was added to each well at 0, 24, 48, and 72 h. The plate was then incubated at 37°C for 2 h. The absorbance was measured at a wavelength of 492 nm using a Spark multimode microplate reader (Tecan).

### 2.6. Flow Cytometry Analysis

The cell apoptosis detection was performed using Annexin V‐FITC/PI (BD Bioscience) double staining reagent. The collected cells were washed twice with PBS and resuspended at a concentration of 1 × 10^6^ cells per milliliter. Under dark conditions, Annexin V‐FITC and PI were added to the cells for apoptosis staining. For cell cycle analysis, the collected cells were again washed twice with PBS and fixed in 70% ethanol solution at 4°C for 24 h. After fixation, the cells were stained with 500 *μ*L of PI (50 *μ*g/mL) reagent for 30 min at room temperature and tested by FCM according to standard procedures.

### 2.7. Extracellular Acidification Rate (ECAR)

The Seahorse XFe96 analyzer (Seahorse Bioscience, Agilent) was used to measure the ECAR. The processed cells were subjected to ECAR analysis using the Seahorse XF Glycolytic Rate Assay Kit (Seahorse Bioscience, Agilent, 103344‐100), following the manufacturer′s instructions.

### 2.8. Quantitative Real‐Time PCR (qRT‐PCR)

Total RNA was extracted using TRIzol reagent (Invitrogen, Carlsbad, CA, United States). The extracted RNA was then reverse‐transcribed into cDNA using the ReverTra Ace qPCR RT Kit (Toyobo, Japan). Subsequently, qRT‐PCR was performed. qRT‐PCR was performed on a Step One Plus real‐time quantitative PCR system using GoTaq qPCR Master Mix (Promega). The expression of the target genes was normalized using *β*‐actin and U6 genes as internal control genes. The relative expression level of RNAs was calculated using the 2^−*Δ* *Δ*CT^ method [15]. The primer sequences were as follows: PMID: 11846609

*β*‐Actin forward primer: 5 ^′^‐TGGCACCCAGCACAATGAA‐3 ^′^; reverse primer: 5 ^′^‐CTAAGTCATAGTCCGCCTAGAAGCA‐3 ^′^.

U6 forward primer: 5 ^′^‐CTCGCTTCGGCAGCACATATACT‐3 ^′^; reverse primer: 5 ^′^‐CGCTTCACGAATTTGCGTGT‐3 ^′^.

LBX2‐AS1 forward primer: 5 ^′^‐AGTTTGTCCCAGGTTTGGCA‐3 ^′^; reverse primer: 5 ^′^‐CATGCCAGGGTCCTTGTTCT‐3 ^′^.

Cyclin D1 forward primer: 5 ^′^‐TCCTACTACCGCCTCACA‐3 ^′^; reverse primer: 5 ^′^‐ACCTCCTCCTCCTCCTCT‐3 ^′^.

HIF‐1*α* forward primer: 5 ^′^‐GAACGTCGAAAAGAAAAGTCTCG‐3 ^′^; reverse primer: 5 ^′^‐CCTTATCAAGATGCGAACTCACA‐3 ^′^.

PKM2 forward primer: 5 ^′^‐ATGTCGAAGCCCCATAGTGAA‐3 ^′^; reverse primer: 5 ^′^‐TGGGTGGTGAATCAATGTCCA‐3 ^′^.

### 2.9. Western Blot Analysis

The cells were treated with cell lysis buffer (Keygen Biotech, China). The supernatant was collected after centrifugation, and the protein concentration was determined. Proteins were then mixed with protein loading buffer and heated onto gels. A total of 50 *μ*g of protein was loaded onto a gel for SDS‐PAGE separation of the total cellular proteins, which were subsequently transferred to a PVDF membrane. The membrane was incubated with 5% nonfat milk and then probed with antibodies overnight at 4°C. The antibodies used were anti‐HIF‐1*α* (1:1000, Abcam), anti‐cyclin D1 (1:1500, CST, United States), anti‐PKM2 (Abcam), and anti‐*β*‐actin (1:2500, Abcam). After overnight incubation, the membrane was washed with TBST and then incubated with a horseradish peroxidase–linked secondary antibody (1:2000) at room temperature for 1 h. The membrane was washed three times with TBST. Finally, the membrane was imaged using the Odyssey dual‐color infrared fluorescence scanning system.

### 2.10. Luciferase Reporter Assay

The 3 ^′^UTR of LBX2‐AS1 and PKM2, which bind to miR‐491‐5p, from the genomic DNA of EC cells was cloned. Then, this sequence is inserted into the pmirGLO control luciferase reporter vector. After incubating for 48 h, the fluorescence activity was detected using the Promega luciferase assay kit according to the manufacturer′s instructions (Shanghai GeneChem, China).

### 2.11. Statistical Analysis

All statistical analyses were performed using SPSS 14.0 software. Quantitative results are presented as mean ± SD. Between‐group statistical analyses were conducted using the Student–Newman–Keuls tests. *p* < 0.05 was considered statistically significant.

## 3. Results

### 3.1. Mechanism of Engendered Radioresistance in EC Cell Lines

To investigate the radioresistant mechanisms of EC cells, we established a radioresistant sub‐cell line (KYSE150R) by exposing EC cells (KYSE150) to a repeated dose of 2 Gy each with 15 rounds of x‐ray irradiation. We irradiated KYSE150 and KYSE150R cells with increasing doses of x‐ray irradiation (0, 3, and 6 Gy) to verify the radioresistant phenotype. KYSE150R cells exhibited a significantly stronger radioresistance at 6 Gy, compared with KYSE150 cells (Figure 1a). Therefore, we selected 6 Gy as the dose of x‐ray irradiation for the subsequent experiments. We then analyzed the differences in metabolites between the KYSE150 and KYSE150R cell lines. The results showed that there were 37 differentially expressed metabolites, including 27 upregulated and 11 downregulated in KYSE150R cells (Figures 1b, 1c, and 1d). We performed partial least squares discriminant analysis (PLS‐DA) on the data to analyze the characteristic metabolites. The results showed that genistein, luteolin, and indoleacrylic could be used as characteristic metabolites (Figure 1e). We then performed a metabolite enrichment analysis, and the results showed that KYSE150 and KYSE150R cells had differences in glucose metabolism pathways (Figure 1f). KEGG pathway results also showed that KYSE150 and KYSE150R cells had differences in glucose metabolism and pyruvate metabolism (Figure 1g).

Figure 1Metabolomics of radioresistant cell lines. (a) Construction of radioresistant cell line (KYSE150). (b) Principal component analysis (PCA) score plot (*n* = 2). (c) Partial least squares discriminant analysis (PLS‐DA) score plot. (d) Differential metabolites in radioresistant cell lines. (e) Variable importance in projection (VIP). (f, g) GO enrichment analysis and KEGG pathway analysis of metabolites.  ^∗^ *p* < 0.05.

**Figure:**
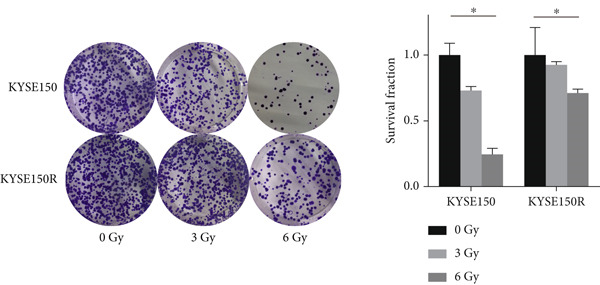
(a)

**Figure:**
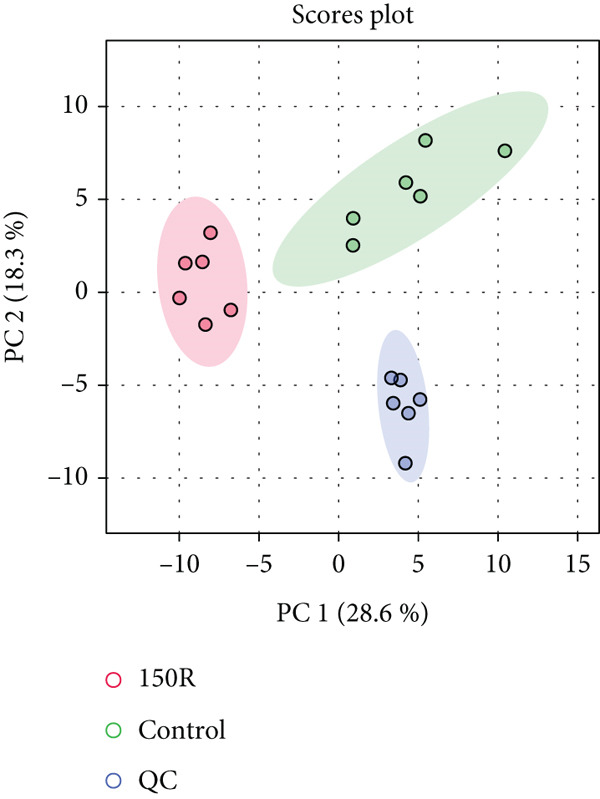
(b)

**Figure:**
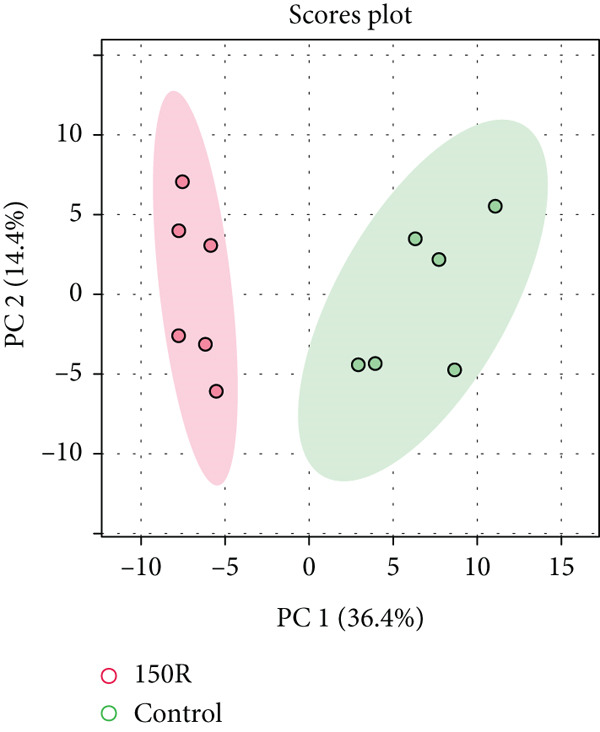
(c)

**Figure:**
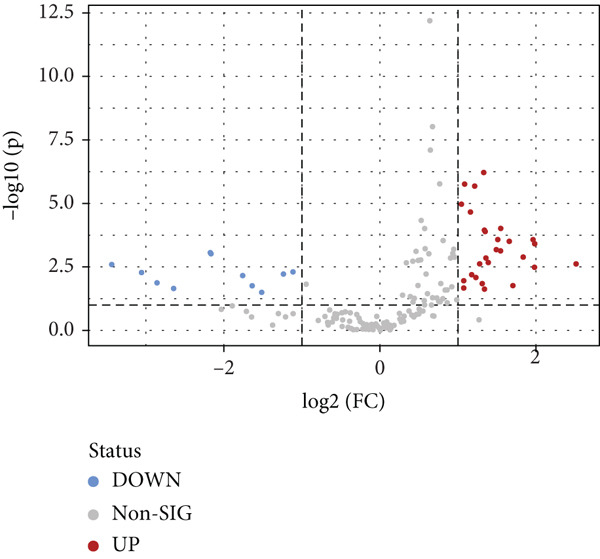
(d)

**Figure:**
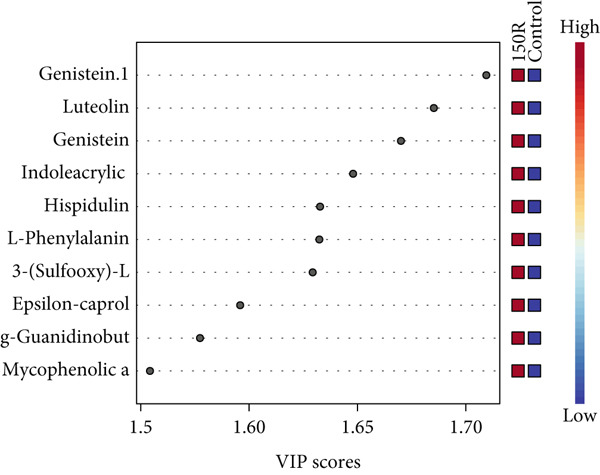
(e)

**Figure:**
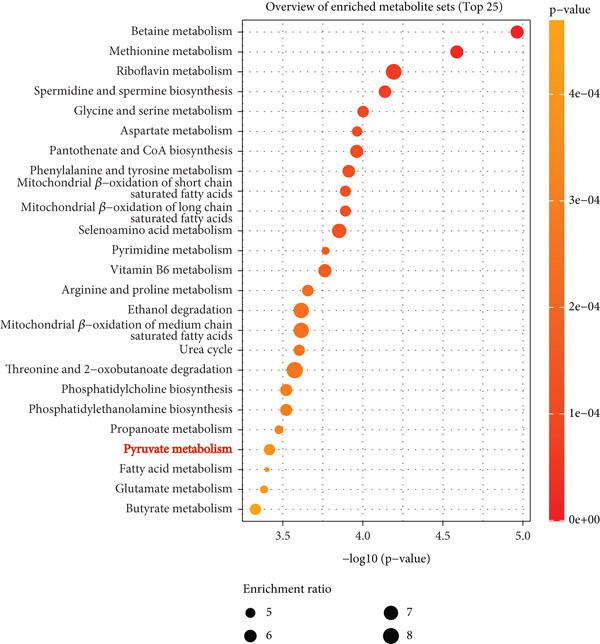
(f)

**Figure:**
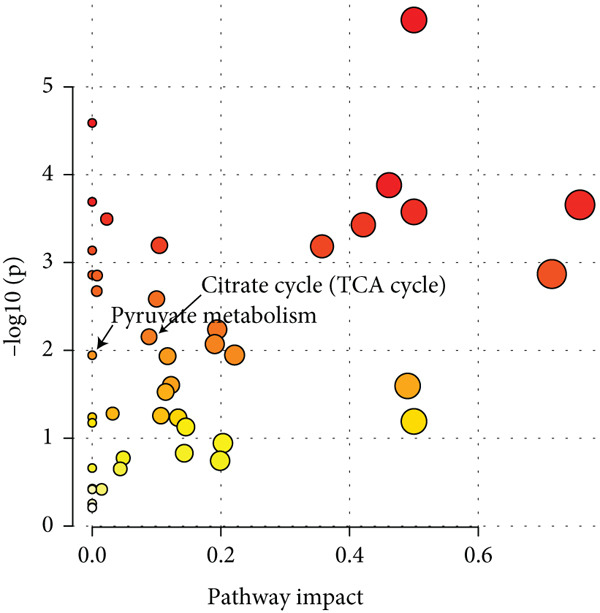
(g)

The radioresistant sub‐cell line changed in terms of cell function was detected. Cell proliferation, cycle, and apoptosis were examined. There was no significant difference in proliferation (Figure 2a) between KYSE150 and KYSE150R cells, but there was a significant difference in cell cycle. The proportion of S phase cells in KYSE150R cells was significantly increased compared with KYSE150 cells (Figure 2b). Cell cycle is an important factor affecting radiotherapy sensitivity, and S phase cells are the least sensitive to x‐ray. The apoptosis rate of cells in both groups was less than 5%, and no significant apoptosis was produced, so the changes in radiotherapy sensitivity are not caused by apoptosis (Figure 2c). The ECAR was detected by seahorse assay to compare the glycolysis level of KYSE150 and KYSE150R cells. The results showed that the glycolysis level of KYSE150R cells was significantly higher than that of KYSE150 cells (Figure 2d). Glycolysis level is an important factor affecting radiotherapy sensitivity. Enhancing glycolysis level could increase radiotherapy resistance. We examined the level of cyclin D1, a key protein involved in the transition of cells from G1 to S phase, and the levels of HIF‐1*α* and PKM2, key glycolysis proteins. The results showed that the levels of cyclin D1, HIF‐1*α*, and PKM2 were significantly increased in KYSE150R cells (Figure 2e,f).

Figure 2The radioresistant sub‐cell line changed in terms of cell function. (a) MTS assay was used to detect cell proliferation. (b) Cell cycle changes were detected by flow cytometry. (c) Flow cytometry was used to detect apoptosis. The apoptosis rates were less than 5%, indicating that there was no obvious apoptosis. (d) The changes of glycolysis were detected by seahorse assay. (e) mRNA levels of cyclin D1, HIF‐1*α*, and PKM2 were detected by qRT‐PCR. (f) Protein levels of cyclin D1, HIF‐1*α*, and PKM2 were detected by Western blot.  ^∗^ *p* < 0.05.

**Figure:**
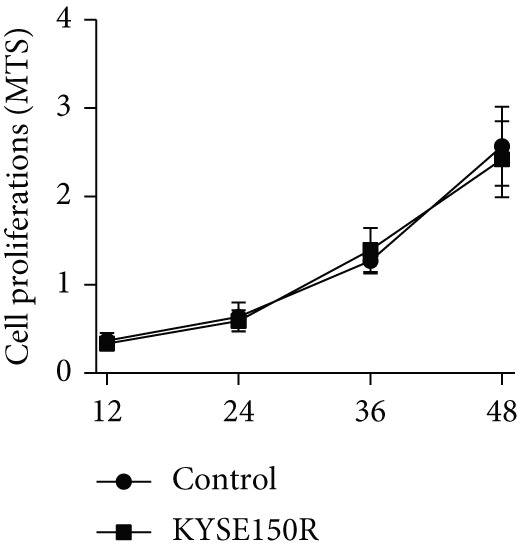
(a)

**Figure:**
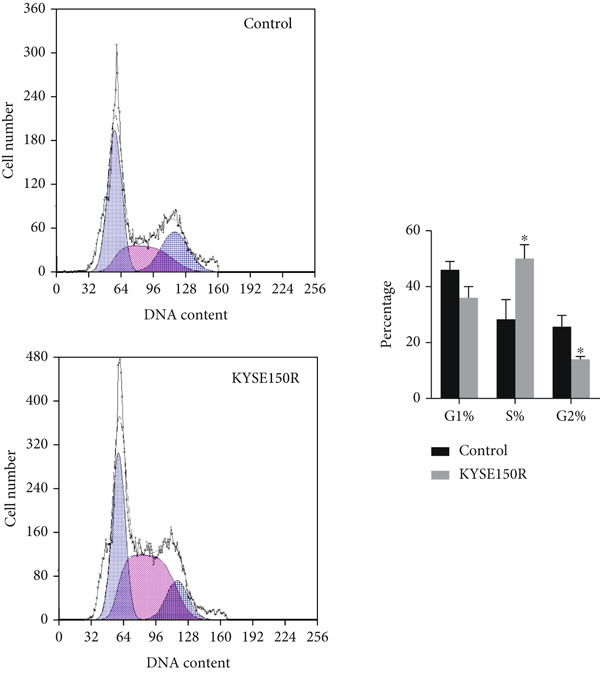
(b)

**Figure:**
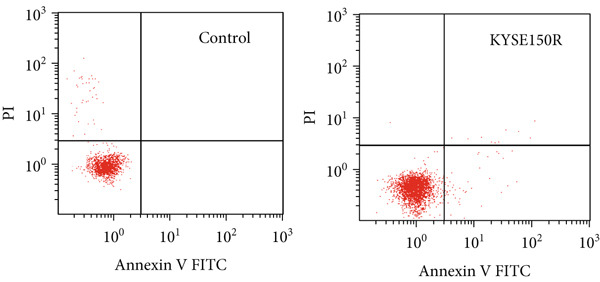
(c)

**Figure:**
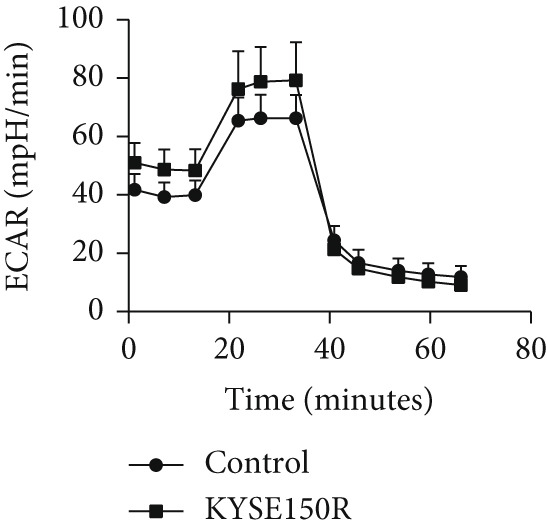
(d)

**Figure:**
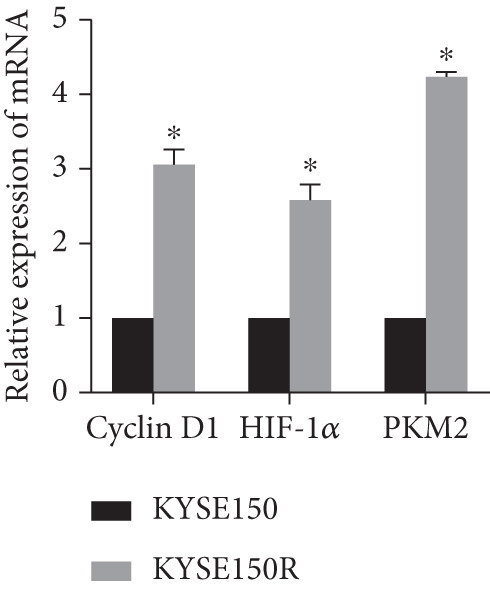
(e)

**Figure:**
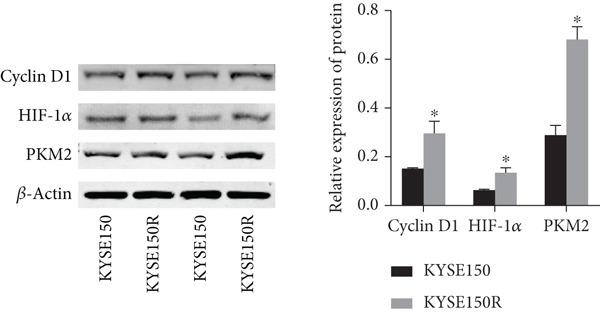
(f)

### 3.2. PKM2 Increased the Levels of LBX2‐AS1, HIF‐1*α*, and Cyclin D1

PKM2 is not only a rate‐limiting enzyme for glycolysis but also directly modulates gene transcription. Studies have demonstrated that PKM2 can act as a histone kinase in the nucleus to upregulate the expression of HIF‐1*α* and cyclin D1. We verified the distribution of PKM2 in cells by immunofluorescence. The results indicated that the level of PKM2 in the nucleus of KYSE150R cells was significantly higher than that of KYSE150 cells (Figure 3a). We constructed a subcellular line with upregulated PKM2 in KYSE150 (Figure 3b). The results revealed that upregulating PKM2 could increase the level of PKM2 in the nucleus (Figure 3a). The mRNA levels of HIF‐1*α* and cyclin D1 were detected, and it was found that upregulating PKM2 significantly increased the mRNA levels of HIF‐1*α* and cyclin D1 (Figure 3c,d). Additionally, we constructed a subcellular line with knocked‐down PKM2 in KYSE150R (Figure 3b). The results showed that the knockdown of PKM2 could reduce the level of PKM2 in the nucleus (Figure 3a) and simultaneously decrease the mRNA levels of HIF‐1*α* and cyclin D1 (Figure 3c,d). These results suggest that levels of HIF‐1*α* and cyclin D1 are regulated by PKM2. We further explored the mechanism underlying the increased level of PKM2 in KYSE150R cells and discovered that LBX2‐AS1 was the key molecule. The expression levels of LBX2‐AS1 in KYSE150 and KYSE150R cells were detected by qRT‐PCR. The results demonstrated that the level of LBX2‐AS1 in KYSE150R cells was significantly higher than that in KYSE150 cells (Figure 3e). Upregulating PKM2 significantly increased the level of LBX2‐AS1, while knocking down PKM2 significantly decreased the level of LBX2‐AS1 (Figure 3e).

Figure 3PKM2 increased the levels of LBX2‐AS1, HIF‐1*α*, and cyclin D1. (a) Immunofluorescence assay was used to detect the distribution of PKM2 in KYSE150 and KYSE150R cells. (b) The levels of PKM2 in each cell were detected by Western blot. (c) qRT‐PCR was used to verify the levels of HIF‐1*α*. (d) qRT‐PCR was used to verify the levels of cyclin D1. (e) qRT‐PCR was used to verify the levels of LBX2‐AS1 by upregulating and knockdown of PKM2.  ^∗^ *p* < 0.05.

**Figure:**
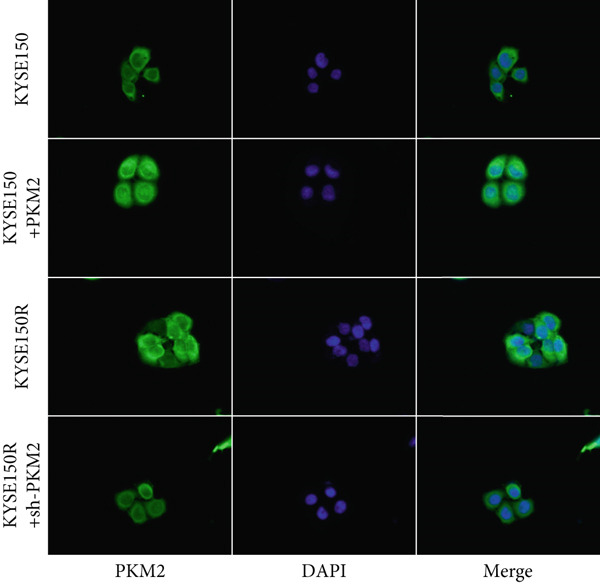
(a)

**Figure:**
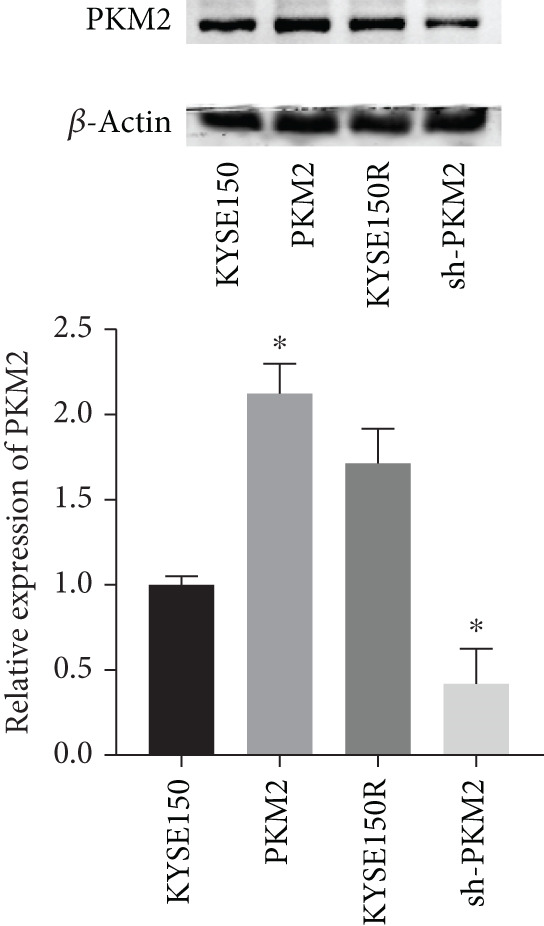
(b)

**Figure:**
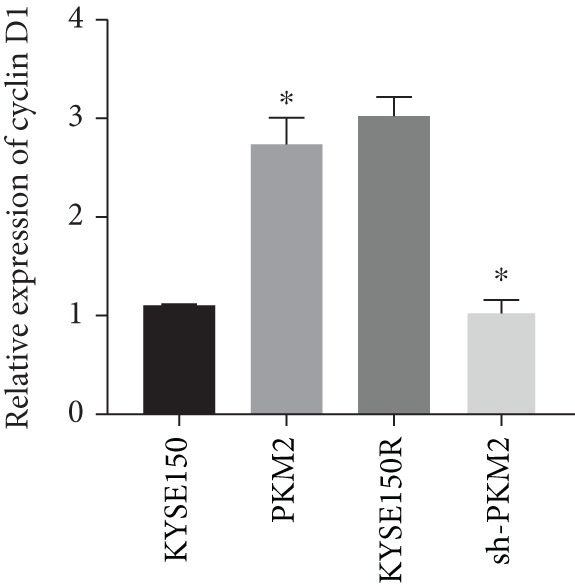
(c)

**Figure:**
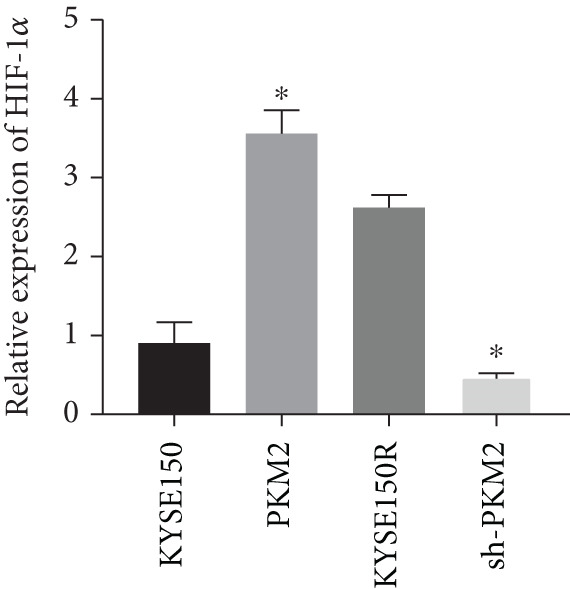
(d)

**Figure:**
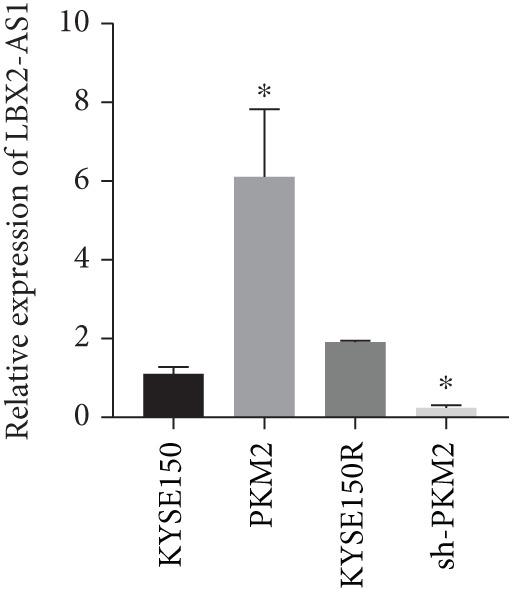
(e)

### 3.3. LBX2‐AS1 Increases PKM2 Levels and Promotes Glycolysis of EC Cells

We constructed sub‐cell lines in KYSE150 and KYSE150R cells with the knockdown of LBX2‐AS1 by lentivirus. The levels of LBX2‐AS1 were detected, and the results showed that compared with the control group, the level of LBX2‐AS1 in the knockdown LBX2‐AS1 sub‐cell lines was significantly reduced, and the effect of LBX2‐AS1a was more significant in both KYSE150 and KYSE150R cells, as the follow‐up experimental group (Figure 4a). Radiotherapy sensitivity was detected by clonogenic assays, and results showed that the knockdown of LBX2‐AS1 significantly reduced the radioresistance both in KYSE150 and KYSE150R cells (Figure 4b). Cell proliferation, cycle, and the level of glycolysis were also examined. The results showed that the knockdown of LBX2‐AS1 significantly reduced the proliferation both in KYSE150 and KYSE150R cells (Figure 4c).The knockdown of LBX2‐AS1 significantly altered the cell cycle and blocked cells in the G1 phase (Figure 4d). It resulted in a significant decrease in the level of glycolysis both in KYSE150 and KYSE150R cells (Figure 4e). Since the knockdown of LBX2‐AS1 retarded the cell cycle in the G1 phase, we further detected cyclin D1, a key protein in the transition from G1 to S phase. The expression of key glycolysis proteins HIF‐1*α* and PKM2 was detected by qRT‐PCR and Western blot. The results showed that compared with the control group, the knockdown of LBX2‐AS1 significantly decreased the expression levels of cyclin D1, HIF‐1*α*, and PKM2 (Figure 4f,g).

Figure 4Function of LBX2‐AS1 in esophageal cancer cell lines. (a) qRT‐PCR was used to verify the knockdown effect of LBX2‐AS1. (b) Cloning assay was used to detect radiotherapy sensitivity. (c) MTS was used to detect the proliferation of esophageal cancer cells. (d) Flow cytometry was used to detect the cycle changes of esophageal cancer cells. (e) Seahorse assay was used to detect changes of glycolysis. (f) Changes of cyclin D1, PKM2, and HIF‐1*α* mRNA expression levels after LBX2‐AS1 knockdown. (g) Changes of cyclin D1, PKM2, and HIF‐1*α* protein expression levels after LBX2‐AS1 knockdown.  ^∗^ *p* < 0.05.

**Figure:**
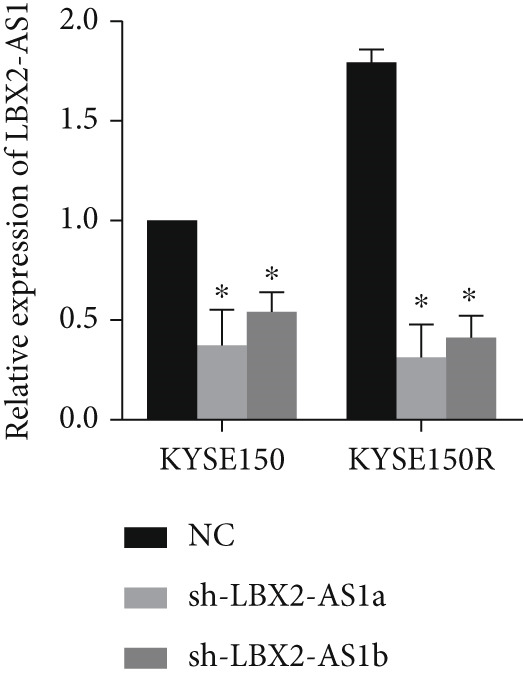
(a)

**Figure:**
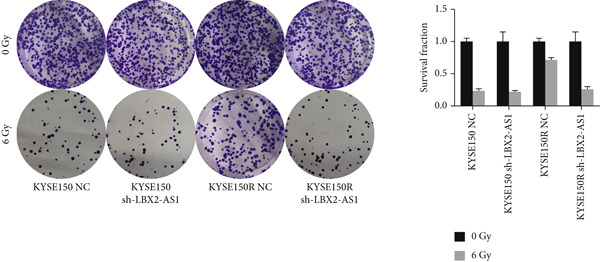
(b)

**Figure:**
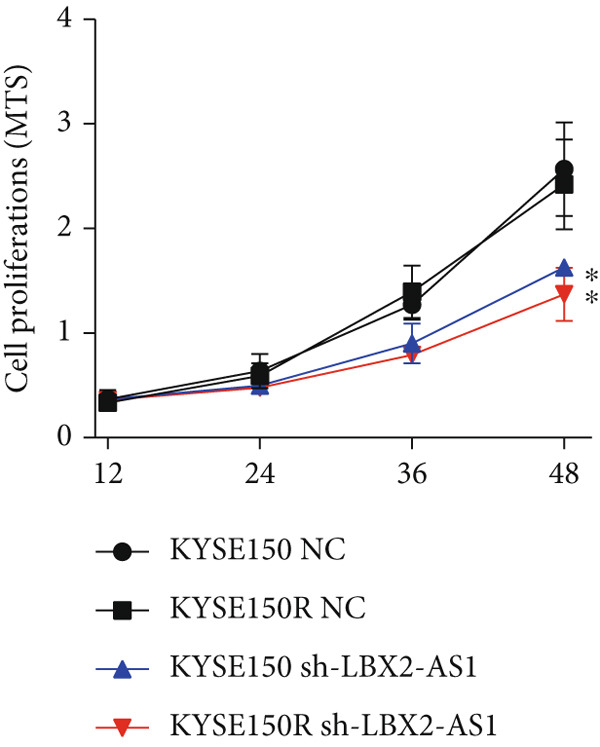
(c)

**Figure:**
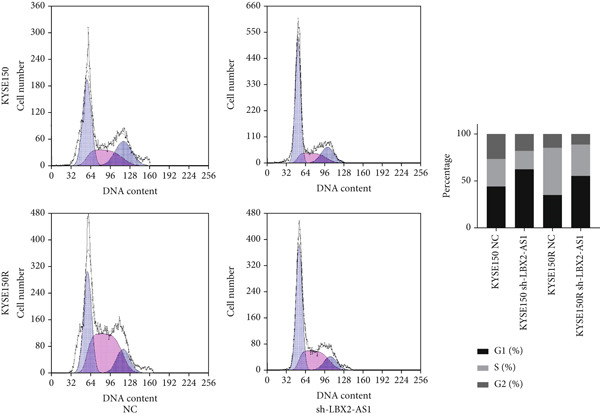
(d)

**Figure:**
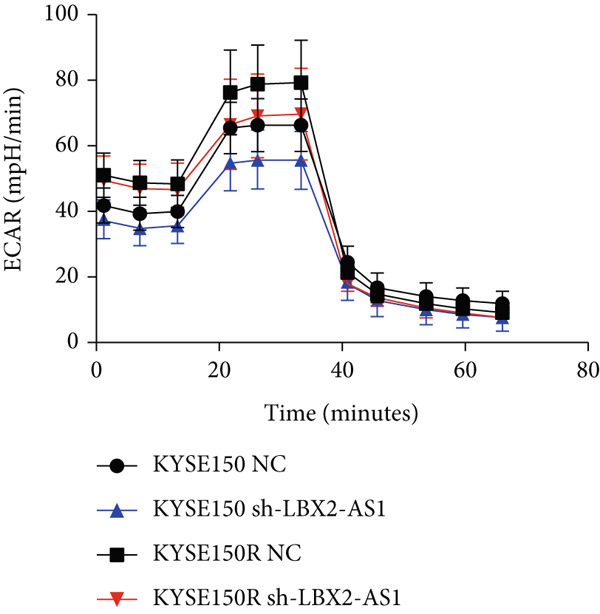
(e)

**Figure:**
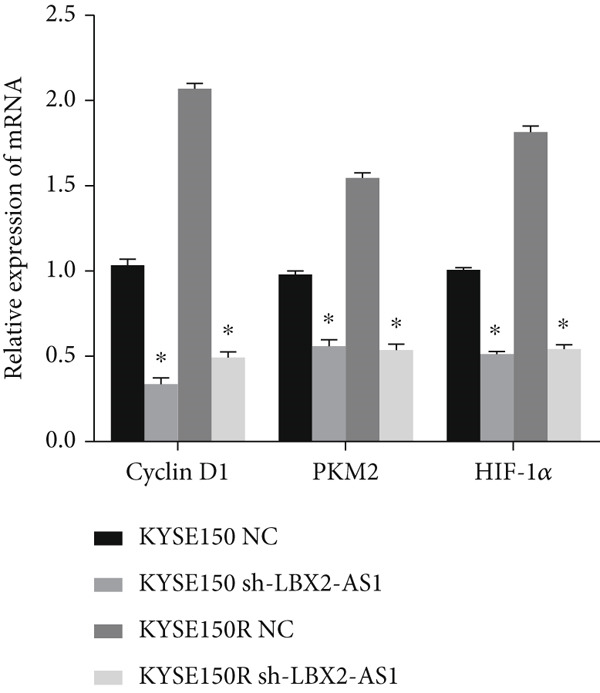
(f)

**Figure:**
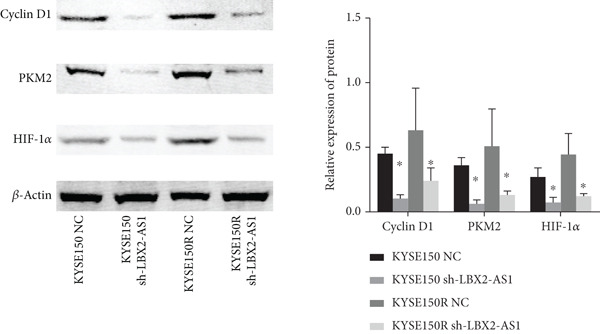
(g)

Figure 5Function of LBX2‐AS1 and miR‐491‐5p in esophageal cancer cell lines. (a) Dual luciferase assay was used to verify the binding of LBX2‐AS1 to miR‐491‐5p. (b) Dual luciferase assay was used to verify that PKM2 mRNA can bind to miR‐491‐5p. (c) Cloning assay was used to detect radiotherapy sensitivity. (d) MTS was used to detect the proliferation of esophageal cancer cells. (e) Flow cytometry was used to detect the cycle changes of esophageal cancer cells. (f) Seahorse assay was used to detect changes of glycolysis.  ^∗^ *p* < 0.05.

**Figure:**
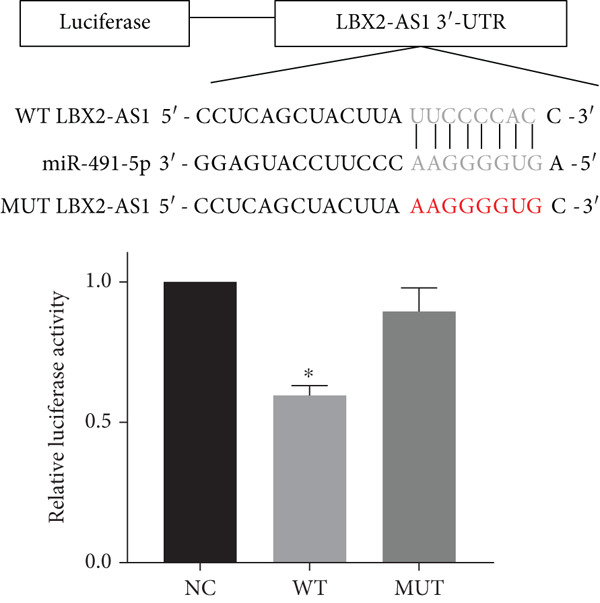
(a)

**Figure:**
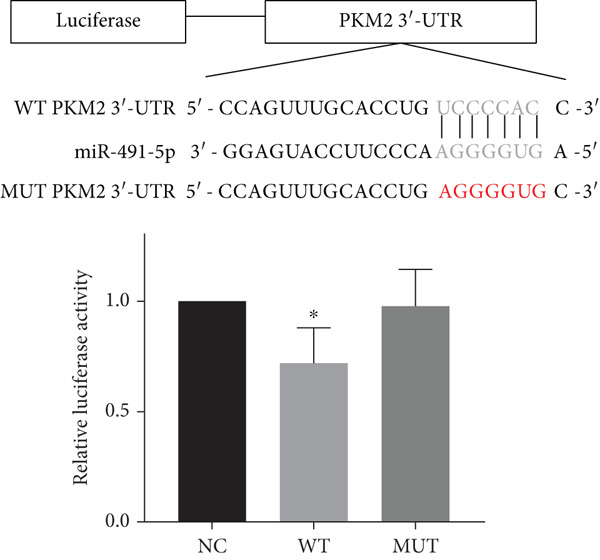
(b)

**Figure:**
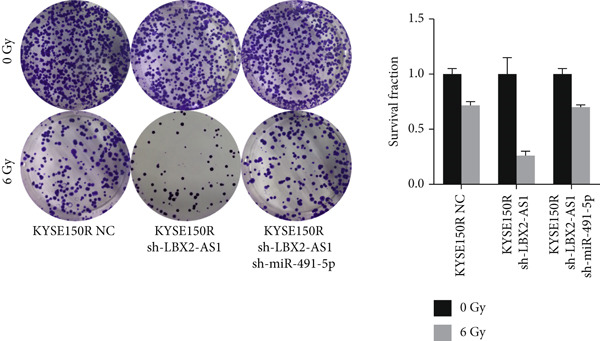
(c)

**Figure:**
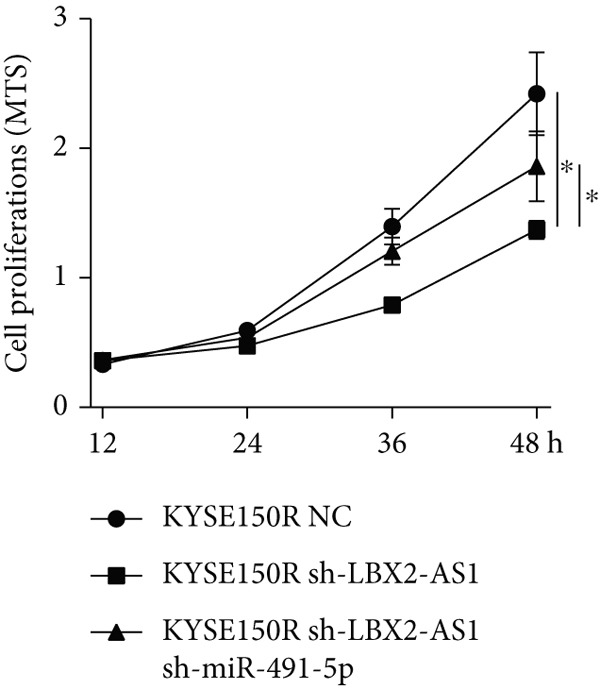
(d)

**Figure:**
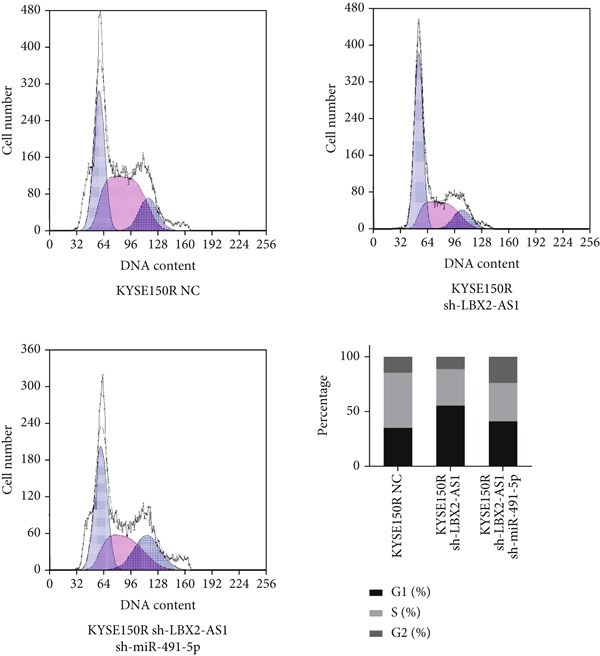
(e)

**Figure:**
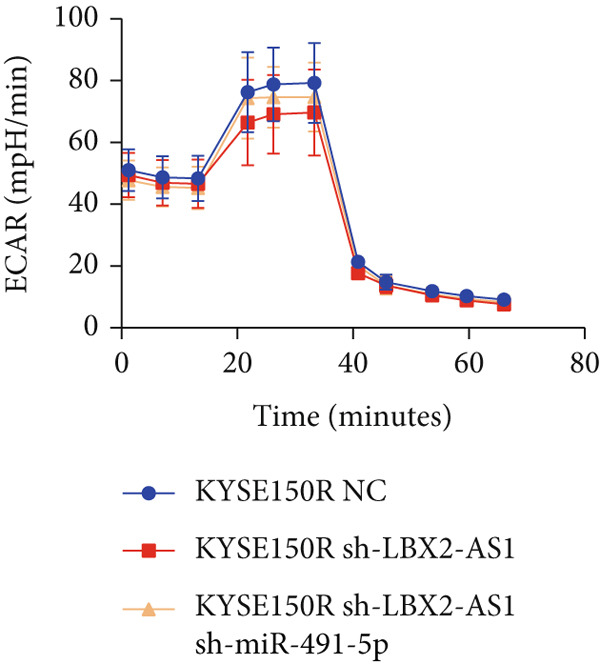
(f)

By searching the database, it was found that PKM2 may be the direct target of LBX2‐AS1. LBX2‐AS1 regulates the expression of PKM2 by adsorbing miR‐491‐5p as a molecular sponge. The dual‐luciferase reporter system was used to verify whether LBX2‐AS1 directly binds to miR‐491‐5p and whether miR‐491‐5p directly binds to the 3 ^′^UTR of PKM2 mRNA. The results showed that the miR‐491‐5p mimic inhibited the luciferase activity of psiCHECK2 containing LBX2‐AS1 (Figure 5a) and PKM2 mRNA (Figure 5b), respectively, compared with the control group, demonstrating that LBX2‐AS1 acts as a molecular sponge by adsorbing miR‐491‐5p to regulate PKM2. We verified that LBX2‐AS1 acts through miR‐491‐5p by response experiments. Plasmid sh‐miR‐491‐5p was added in KYSE150R cells while knockdown of LBX2‐AS1, and the radiosensitivity was detected by clonogenic assays. The results showed that sh‐miR‐491‐5p could increase the radioresistance of KYSE150R cells and reverse the effect of downregulated LBX2‐AS1 on radiotherapy sensitivity (Figure 5c). Cell proliferation, cycle, and glycolysis levels were also measured. The results showed that sh‐miR‐491‐5p significantly increased the proliferation of KYSE150R cells (Figure 5d), altered the cell cycle (Figure 5e), and enhanced glycolysis (Figure 5f). sh‐miR‐491‐5p reverted the changes caused by downregulated LBX2‐AS1.

### 3.4. LBX2‐AS1 Is Overexpressed in EC Tissues and Associated With Radiotherapy Sensitivity

We explored the expression levels of LBX2‐AS1 through the BEST website (https://rookieutopia.com/app_direct/BEST/#PageHomeAnalysisModuleSelection). The data showed that the expression level of LBX2‐AS1 in EC tissues was significantly higher than that in adjacent tissues (Figure 6a). We also investigated the association of LBX2‐AS1 with age, tumor location, and lymph node metastasis. The results showed that the expression level of LBX2‐AS1 was higher in the higher age group (Figure 6b) and higher in the lower segment (Figure 6c) of EC. The expression level of LBX2‐AS1 is correlated with EC metastasis, and the higher the degree of regional lymph node metastasis, the higher the expression level of LBX2‐AS1 (Figure 6d).

Figure 6Relationship between the expression level of LBX2‐AS1 and esophageal cancer. (a) LBX2‐AS1 expression levels in esophageal cancer tissues in GSE130078, GSE53622, GSE53624, and TCGA data; (b) relationship between LBX2‐AS1 and age; (c) relationship between LBX2‐AS1 and tumor location; (d) relationship between LBX2‐AS1 and lymphatic metastasis; (e) overall survival in patients with the level of LBX2‐AS1 in serum; (f) progression‐free survival in patients with the level of LBX2‐AS1 in serum.

**Figure:**
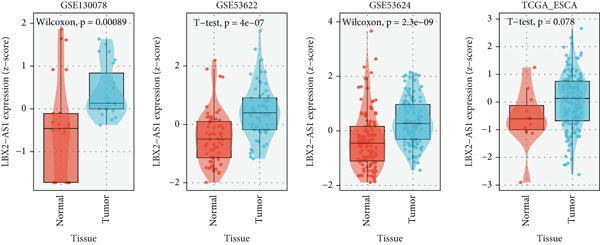
(a)

**Figure:**
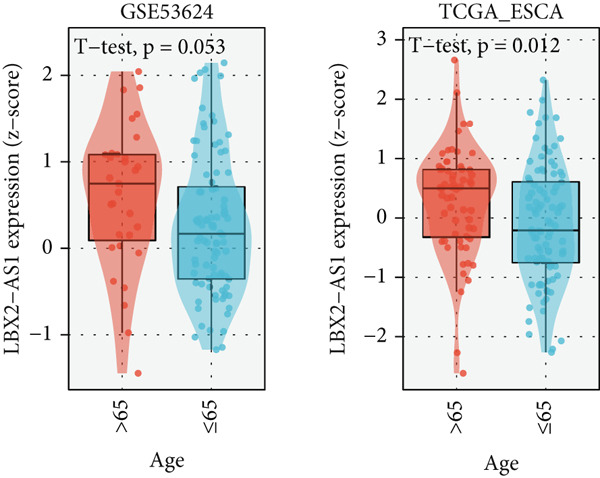
(b)

**Figure:**
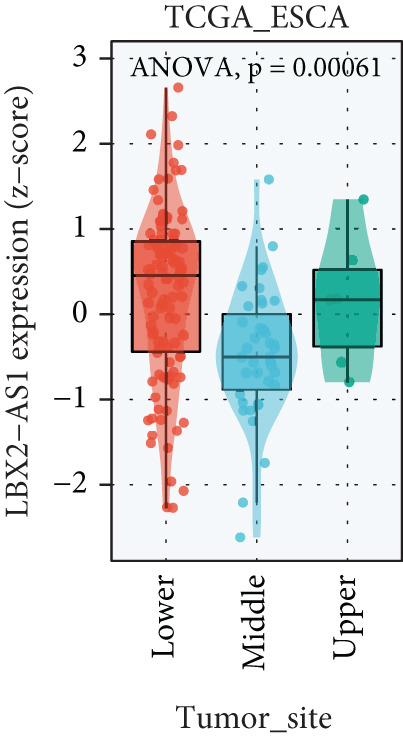
(c)

**Figure:**
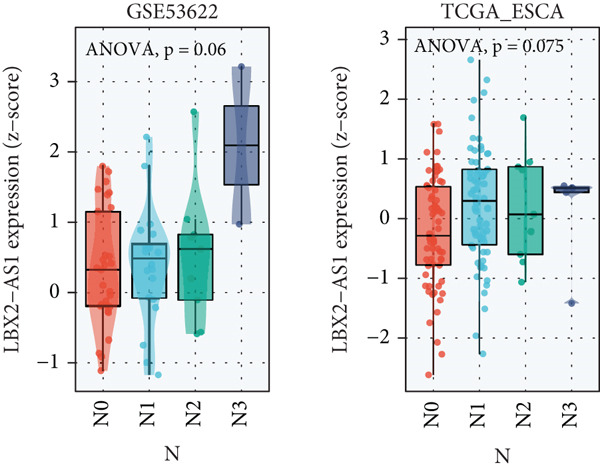
(d)

**Figure:**
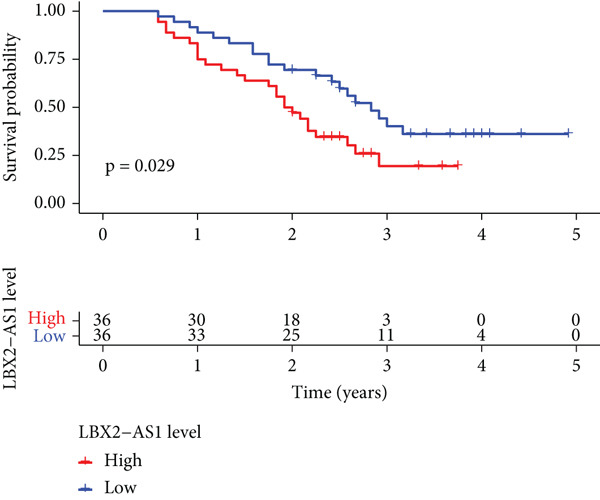
(e)

**Figure:**
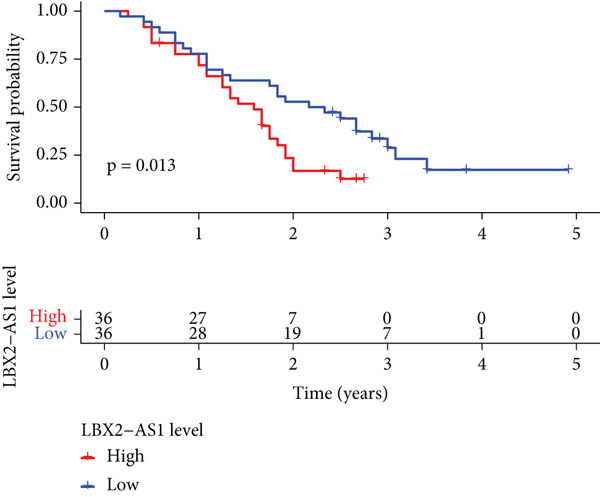
(f)

Public databases showed that expression levels of LBX2‐AS1 were correlated with the progression of EC. We further investigated the relationship between the expression level of LBX2‐AS1 and the characteristics of patients treated with radiotherapy for EC. We collected serum samples from 72 patients treated with radiotherapy for EC, and the levels of LBX2‐AS1 in the serum of patients were separated into groups based on the relative levels. The results showed that the expression level of LBX2‐AS1 was correlated with the disease control rate (DCR) (*p* = 0.003). The levels of LBX2‐AS1 in stable disease (SD) patients′ serum were significantly higher than those in partial response (PR) patients. Patients with high level of LBX2‐AS1 were more likely to have lymphatic metastasis (Table 1). Patients with ESCC with low expression of LBX2‐AS1 had better overall survival and progression‐free survival compared with those with high expression of LBX2‐AS1 (Figure 6e,f).

**Table 1:** Association of LBX2‐AS1 in cancer tissues with patient characteristics.

**Factor**	**High LBX2-AS1 (** **n** = 36**)**		**Low LBX2-AS1 (** **n** = 36**)**		**χ** ^2^	**p**
	**N**	**%**	**N**	**%**		
Gender					0.000	1.000
Male	24	66.7	24	66.7		
Female	12	33.3	12	33.3		
Clinical stage					0.306	0.858
I	3	8.3	3	8.3		
II	23	63.9	25	69.4		
III	10	27.8	8	22.2		
Lymphatic					5.675	0.017
Yes	11	30.6	3	8.3		
No	25	69.4	33	91.7		
Smoking					0.520	0.471
Yes	16	44.4	13	36.1		
No	20	55.6	23	63.9		
DCR					9.000	0.003
CR	18	50.0	30	83.3		
PR	18	50.0	6	16.7		

## 4. Discussion

The principle of radiotherapy is to inhibit the proliferation of cancer cells by damaging the chromosomes, causing DNA damage and inducing apoptosis of cells, based on the energy transmitted [16]. During radiation therapy, radiation could induce radiation resistance [17]. The subsets of cells that eventually dominated showed resistance to radiation and had a stronger malignant phenotype than the original parent cells [18]. As a result, patients often have a poor prognosis. At the same time, the effect of radiation therapy is limited by the radiation dose, which should be effective in killing cancer cells without damaging normal cells. Therefore, we cannot rely on increasing the radiation dose to improve the effectiveness of radiation therapy. It is important to study the mechanism of radiotherapy resistance to improve the therapeutic effect of radiotherapy.

To investigate the radioresistant mechanisms of EC cells, we established radioresistant sub‐cell lines. We found that the engendered radioresistance is closely related to glycolysis by detecting metabolites and analyzing signaling pathways. We also examined the factors related to radiotherapy sensitivity, such as the cell cycle and apoptosis, and found that the cell cycle of radioresistant sub‐cell lines changed significantly, but apoptosis did not change significantly. These results suggested that changes in glycolysis and the cell cycle are key factors in engendered radioresistance. Glycolysis could produce a large number of metabolites, such as pyruvate, lactate, glutathione, and NAD(P)H/NAD(P)+, while producing a large amount of ATP. These metabolites could resist the oxidative stress caused by radiation therapy and constitute a network of REDOX buffers in tumor cells, which effectively remove free radicals and ROS, thus weakening the efficacy of radiation therapy [19, 20]. The cell cycle is recognized as an important factor affecting radiotherapy sensitivity. S phase cells were the least sensitive to radiation, and G2 phase cells were the most sensitive to radiation. The proportion of S phase cells increased, and G2 phase cells decreased in the radioresistant sub‐cell line constructed in this study, which was consistent with this characteristic. Characteristics of G1 phase cells are active metabolism and rapid synthesis of RNA and protein, which require a lot of energy. Enhanced glycolysis could provide a lot of energy to promote G1 phase cells to enter S phase. This explains the relationship between increased glycolysis and cell cycle change in the radioresistant sub‐cell line.

Considering the safety and efficacy in the clinical treatment process [21], lncRNA has characteristics of easy extraction, high sensitivity and specificity, and is considered a potential novel biomarkers for cancer diagnosis, treatment, and prognosis. With the development of high‐throughput sequencing, many public databases (e.g., TCGA and GEO) have been used to predict and identify valuable lncRNAs widely [22]. In this study, we first analyzed public databases and found that lncRNA LBX2‐AS1 was highly expressed in EC tissues and correlated with the progression of EC. Studies have shown that LBX2‐AS1 was detectable and stable in serum samples [23] and is a good biomarker for patients with radiation therapy who cannot be surgically resected. We further analyzed the relationship between the expression level of LBX2‐AS1 in serum of patients with radiotherapy for EC and DCR and prognosis and found that LBX2‐AS1 could be used as a biomarker of DCR and prognosis.

We detected the expression of LBX2‐AS1 in the radioresistant sub‐cell line, and the results showed that the expression of LBX2‐AS1 increased, which was consistent with the results of clinical specimens, indicating that the expression level of LBX2‐AS1 was related to radiotherapy sensitivity. Next, we demonstrated the role of LBX2‐AS1 in EC cells, and results showed that the knockdown of LBX2‐AS1 significantly reduced the proliferation and glycolysis of EC cells and blocked cells in G1 phase. We investigated the molecular mechanism and found that the knockdown of LBX2‐AS1 reduces the expression levels of cyclin D1, HIF‐1*α*, and PKM2 by targeting PKM2. PKM2 mainly exists in two conformations within cells: dimer and tetramer. PKM2 dimer mainly regulates gene expression in the cell nucleus. The PKM2 tetramer mainly catalyzes the formation of pyruvate and promotes glycolysis in the cytoplasm [24]. HIF‐1*α* is the key glycolytic protein, and the expression level of HIF‐1*α* was influenced by the expression level of PKM2 [25]. LBX2‐AS1 regulates the expression of HIF‐1*α* by targeting PKM2, which explains the mechanism of inhibiting glycolysis by knocking down LBX2‐AS1. Reduced glycolysis levels decrease energy supply and downregulate the synthesis of cyclin D1, a key protein that promotes cell transition from G1 phase to S phase. This explains the mechanism by which the knockdown of LBX2‐AS1 increases the proportion of G1 phase cells and inhibits cyclin D1 expression.

Our results demonstrate that overexpression of PKM2 upregulated LBX2‐AS1 expression level. Knocking down PKM2 can reduce the expression level of LBX2‐AS1 in radioresistant cell lines. PKM2 acts as a transcription factor in the nucleus. PKM2 could upregulate the expression of HIF‐1*α* and c‐Myc by binding to the promoter region [26, 27]. Therefore, we examined the distribution of PKM2 in radioresistant and control cell lines. We found that the level of PKM2 in the nucleus increased significantly in radioresistant cell lines, corresponding to the increase of LBX2‐AS1 level. The knockdown of PKM2 in radioresistant cell lines significantly reduced the level of PKM2 in the nucleus, corresponding to a decrease in the level of LBX2‐AS1. Enhanced glycolysis and overexpressed PKM2 in radioresistant cell lines may explain the increased expression level of LBX2‐AS1. Therefore, the generation of radioresistance in EC cells may be related to the LBX2‐AS1/miR‐491‐5p/PKM2 positive feedback loop. Our study demonstrated that serum LBX2‐AS1 expression levels in patients with EC could indicate DCR and prognosis. It is a good biomarker for radiotherapy patients who cannot obtain tissue samples. Radioresistance with high expression of LBX2‐AS1 may be caused by increased glycolysis. Corresponding measures could be taken to increase the sensitivity of radiotherapy.

This study is subject to certain limitations. The function of LBX2‐AS1 in EC animal models needs to be further explored. In addition, the potential of LBX2‐AS1 as a target to increase radiotherapy sensitivity in EC needs to be confirmed by further studies.

## Ethics Statement

The protocol was approved by the Ethics Committee of the Fourth Hospital of Hebei Medical University (2023KY027) in accordance with the Declaration of Helsinki.

## Conflicts of Interest

The authors declare no conflicts of interest.

## Funding

No funding was received for this manuscript.

## Data Availability

The data that support the findings of this study are available from the corresponding author upon reasonable request.

## References

[1] Sung H. , Ferlay J. , Siegel R. L. , Laversanne M. , Soerjomataram I. , Jemal A. , and Bray F. , Global Cancer Statistics 2020: Globocan Estimates of Incidence and Mortality Worldwide for 36 Cancers in 185 Countries, CA: A Cancer Journal for Clinicians. (2021) 71, no. 3, 209–249, 10.3322/caac.21660, 33538338. 33538338

[2] Barsouk A. , Rawla P. , Hadjinicolaou A. V. , Aluru J. S. , and Barsouk A. , Targeted Therapies and Immunotherapies in the Treatment of Esophageal Cancers, Medical Sciences. (2019) 7, no. 10, 10.3390/medsci7100100, 31561465. PMC6836115 31561465

[3] Zheng H. , Ren W. , Pan X. , Zhang Q. , Liu B. , Liu S. , He J. , and Zhou Z. , Role of Intravoxel Incoherent Motion MRI in Early Assessment of the Response of Esophageal Squamous Cell Carcinoma to Chemoradiotherapy: A Pilot Study, Journal of Magnetic Resonance Imaging. (2018) 48, no. 2, 349–358, 10.1002/jmri.25934, 2-s2.0-85040004077, 29297204. 29297204

[4] Parisi E. , Genestreti G. , Sarnelli A. , Ghigi G. , Arpa D. , Burgio M. A. , Gavelli G. , Rossi A. , Scarpi E. , Monti M. , Tesei A. , Polico R. , and Romeo A. , Accelerated Hypofractionated Radiotherapy Plus Chemotherapy for Inoperable Locally Advanced Non-Small-Cell Lung Cancer: Final Results of a Prospective Phase-II Trial With a Long-Term Follow-Up, Radiation Oncology. (2019) 14, no. 1, 10.1186/s13014-019-1317-x, 2-s2.0-85068162944, 31234868. PMC6591967 31234868

[5] Nakajima M. and Kato H. , Treatment Options for Esophageal Squamous Cell Carcinoma, Expert Opinion on Pharmacotherapy. (2013) 14, no. 10, 1345–1354, 10.1517/14656566.2013.801454, 2-s2.0-84878886294. 23675862

[6] Wu C. C. and Chen C. J. , Esophageal Carcinoma, New England Journal of Medicine. (2015) 372, no. 15, 10.1056/NEJMc1500692, 2-s2.0-84927164937, 25853761. 25853761

[7] Liberti M. V. and Locasale J. W. , The Warburg Effect: How Does It Benefit Cancer Cells?, Trends in Biochemical Sciences. (2016) 41, no. 3, 211–218, 10.1016/j.tibs.2015.12.001, 2-s2.0-84959451365, 26778478. 26778478 PMC4783224

[8] Yang Y. , Chong Y. , Chen M. , Dai W. , Zhou X. , Ji Y. , Qiu G. , and Du X. , Targeting Lactate Dehydrogenase A Improves Radiotherapy Efficacy in Non-Small Cell Lung Cancer: From Bedside to Bench, Journal of Translational Medicine. (2021) 19, no. 1, 10.1186/s12967-021-02825-2, 33902615. PMC8074241 33902615

[9] Sattler U. G. , Meyer S. S. , Quennet V. , Hoerner C. , Knoerzer H. , Fabian C. , Yaromina A. , Zips D. , Walenta S. , Baumann M. , and Mueller-Klieser W. , Glycolytic Metabolism and Tumour Response to Fractionated Irradiation, Radiotherapy and Oncology. (2010) 94, no. 1, 102–109, 10.1016/j.radonc.2009.11.007, 2-s2.0-74549186565, 20036432. 20036432

[10] Hon C. C. , Ramilowski J. A. , Harshbarger J. , Bertin N. , Rackham O. J. , Gough J. , Denisenko E. , Schmeier S. , Poulsen T. M. , Severin J. , Lizio M. , Kawaji H. , Kasukawa T. , Itoh M. , Burroughs A. M. , Noma S. , Djebali S. , Alam T. , Medvedeva Y. A. , Testa A. C. , Lipovich L. , Yip C. W. , Abugessaisa I. , Mendez M. , Hasegawa A. , Tang D. , Lassmann T. , Heutink P. , Babina M. , Wells C. A. , Kojima S. , Nakamura Y. , Suzuki H. , Daub C. O. , de Hoon M. J. , Arner E. , Hayashizaki Y. , Carninci P. , and Forrest A. R. , An Atlas of Human Long Non-Coding RNAs With Accurate 5′ Ends, Nature. (2017) 543, no. 7644, 199–204, 10.1038/nature21374, 2-s2.0-85015225394, 28241135. 28241135 PMC6857182

[11] Neumann P. , Jae N. , Knau A. , Glaser S. F. , Fouani Y. , Rossbach O. , Krüger M. , John D. , Bindereif A. , Grote P. , Boon R. A. , and Dimmeler S. , The LncRNA GATA6-AS Epigenetically Regulates Endothelial Gene Expression via Interaction With LOXL2, Nature Communications. (2018) 9, no. 1, 10.1038/s41467-017-02431-1, 2-s2.0-85040814071, 29339785. PMC5770451 29339785

[12] Garcia-Padilla C. , Aranega A. , and Franco D. , The Role of Long Non-Coding RNAs in Cardiac Development and Disease, AIMS Genetics. (2018) 5, no. 2, 124–140, 10.3934/genet.2018.2.124, 31435517. 31435517 PMC6698576

[13] Chi H. C. , Tsai C. Y. , Tsai M. M. , Yeh C. T. , and Lin K. H. , Roles of Long Noncoding RNAs in Recurrence and Metastasis of Radiotherapy-Resistant Cancer Stem Cells, International Journal of Molecular Sciences. (2017) 18, no. 9, 10.3390/ijms18091903, 2-s2.0-85029155178, 28872613. PMC5618552 28872613

[14] Zhang Y. , Chen W. , Pan T. , Wang H. , Zhang Y. , and Li C. , LBX2-AS1 Is Activated by ZEB1 and Promotes the Development of Esophageal Squamous Cell Carcinoma by Interacting With HNRNPC to Enhance the Stability of ZEB1 and ZEB2 mRNAs, Biochemical and Biophysical Research Communications. (2019) 511, no. 3, 566–572, 10.1016/j.bbrc.2019.02.079, 2-s2.0-85061973793, 30824187. 30824187

[15] Livak K. J. and Schmittgen T. D. , Analysis of Relative Gene Expression Data Using Real-Time Quantitative PCR and the 2−*ΔΔ*CT Method, Methods. (2001) 25, no. 4, 402–408, 10.1006/meth.2001.1262, 2-s2.0-0035710746. 11846609

[16] Krause M. , Dubrovska A. , Linge A. , and Baumann M. , Cancer Stem Cells: Radioresistance, Prediction of Radiotherapy Outcome and Specific Targets for Combined Treatments, Advanced Drug Delivery Reviews. (2017) 109, 63–73, 10.1016/j.addr.2016.02.002, 2-s2.0-84958559501, 26877102. 26877102

[17] Jin J. , Guo Y. , Dong X. , Liu J. , and He Y. , Methylation-Associated Silencing of mir-193b Improves the Radiotherapy Sensitivity of Esophageal Cancer Cells by Targeting Cyclin D1 in Areas With Zinc Deficiency, Radiotherapy and Oncology. (2020) 150, 104–113, 10.1016/j.radonc.2020.06.022, 32580002. 32580002

[18] Park S. Y. , Lee C. J. , Choi J. H. , Kim J. H. , Kim J. W. , Kim J. Y. , and Nam J. S. , The JAK2/STAT3/CCND2 Axis Promotes Colorectal Cancer Stem Cell Persistence and Radioresistance, Journal of Experimental & Clinical Cancer Research. (2019) 38, no. 1, 10.1186/s13046-019-1405-7, 2-s2.0-85072101764, 31511084. PMC6737692 31511084

[19] Kobliakov V. A. , The Mechanisms of Regulation of Aerobic Glycolysis (Warburg Effect) by Oncoproteins in Carcinogenesis, Biochemistry (Moscow). (2019) 84, no. 10, 1117–1128, 10.1134/S0006297919100018, 2-s2.0-85073426218, 31694508. 31694508

[20] Shen H. , Hau E. , Joshi S. , Dilda P. J. , and Mcdonald K. L. , Sensitization of Glioblastoma Cells to Irradiation by Modulating the Glucose Metabolism, Molecular Cancer Therapeutics. (2015) 14, no. 8, 1794–1804, 10.1158/1535-7163.MCT-15-0247, 2-s2.0-84941899578, 26063767. 26063767

[21] Shi Y. , Song H. , Liu J. , Lin J. , and Fang L. , Comprehensive Evaluation of Clinical Application of Balanced Compound Amino Acid Injection, Frontiers in Nutrition. (2022) 9, 880256, 10.3389/fnut.2022.880256, 35719164. 35719164 PMC9203120

[22] Wang Y. , Nie H. , He X. , Liao Z. , Zhou Y. , Zhou J. , and Ou C. , The Emerging Role of Super Enhancer-Derived Noncoding RNAs in Human Cancer, Theranostics. (2020) 10, no. 24, 11049–11062, 10.7150/thno.49168, 33042269. 33042269 PMC7532672

[23] Li Q. , Xie H. , Jin Z. , Huang J. , Wang S. , and Zhang Z. , Overexpression of Long Noncoding RNA LBX2-AS1 Promotes the Proliferation of Colorectal Cancer, Technology in Cancer Research & Treatment. (2021) 20, 1080265477, 10.1177/1533033821997829. PMC7983235 33733923

[24] Wu B. , Liang Z. , Lan H. , Teng X. , and Wang C. , The Role of PKM2 in Cancer Progression and Its Structural and Biological Basis, Journal of Physiology and Biochemistry. (2024) 80, no. 2, 261–275, 10.1007/s13105-024-01007-0, 38329688. 38329688

[25] Azoitei N. , Becher A. , Steinestel K. , Rouhi A. , Diepold K. , Genze F. , Simmet T. , and Seufferlein T. , PKM2 Promotes Tumor Angiogenesis by Regulating HIF-1*α* Through NF-*κ*B Activation, Molecular Cancer. (2016) 15, no. 1, 10.1186/s12943-015-0490-2, 2-s2.0-84953277838, 26739387. PMC4704385 26739387

[26] Li X. , Zhang Y. , Wang X. , Lin F. , Cheng X. , Wang Z. , and Wang X. , Long Non-Coding RNA CTSLP8 Mediates Ovarian Cancer Progression and Chemotherapy Resistance by Modulating Cellular Glycolysis and Regulating c-Myc Expression Through PKM2, Cell Biology and Toxicology. (2022) 38, no. 6, 1027–1045, 10.1007/s10565-021-09650-9, 34510316. 34510316 PMC9750935

[27] Wang Y. , Lian H. , Li J. , Zhao M. , Hao Z. , Zheng X. , Zhao L. , and Cui J. , The HIF-1*α*/PKM2 Feedback Loop in Relation to EGFR Mutational Status in Lung Adenocarcinoma, Journal of Investigative Surgery. (2024) 37, no. 1, 2301081, 10.1080/08941939.2023.2301081, 38224012. 38224012

